# Positive impact of nutrition in the prevention of peripheral vascular disease and severe acute respiratory syndrome coronavirus 2: review

**DOI:** 10.3389/fnut.2024.1418028

**Published:** 2024-09-19

**Authors:** Zubair A. Karim, Rebecca A. Reese, Adrianne N. Smith, Madeline E. Blackadar, Vishal Arora, Nicole M. Moore, Emily A. Johnson

**Affiliations:** ^1^Department of Nutrition and Dietetics, College of Allied Health Sciences, Augusta University, Augusta, GA, United States; ^2^Department of Medicine: Cardiology, Wellstar MCG Health, Augusta University, Augusta, GA, United States

**Keywords:** severe acute respiratory syndrome coronavirus 2, peripheral vascular disease, medical nutrition therapy, Western Dietary, physical activity

## Abstract

Recent research has shown that there is a link between the trend of cardiovascular disease (CVD), chronic symptoms of SARS-CoV-2 (COVID-19), and medical nutrition therapy. Making positive changes to an individual’s lifestyle can help to reduce the symptoms that follow exposure to CVD and COVID-19. Sustainable nutrition and lifestyle changes can positively impact an individual’s health. Studies have considered the risk factors associated with the disease, medical history, the link between nutrition and peripheral vascular disease (PVD), symptom management, and the interrelationship between nutrition, COVID-19, and PVD. One study has demonstrated that Western Dietary intake can boost the innate immune system while suppressing humoral response, causing chronic inflammation and poor host defense against viruses. However, further investigation is needed to confirm. Patients with PVD and COVID-19 have experienced a reduction in side effects when prescribed a regimen of medical nutrition therapy, heart-healthy diets, and adequate physical activity before and after symptoms of both diseases appear. This approach has proven to be a protective factor during the combination of both illnesses. Our findings indicate that balanced diet and lifestyle are essential in supporting an optimal immune system that can reduce the risk of virus load in individuals at risk of infection and symptoms from COVID-19 and PVD.

## What is cardiovascular disease?

Cardiovascular disease (CVD) is a class of diseases that involves the heart or blood vessels and comprises many different types of conditions, i.e., heart disease, heart attack, stroke, heart failure, arrhythmia, coronary artery disease, or heart valve problems. Some of these might develop simultaneously or may lead to other physiological complications. It should be noted that age is one of the major risk factors associated with this disease. Cardiovascular disease can be related to changes in circulatory blood flow, causing narrowing of arterial/venous blood vessels and causing poor blood flow to the other part of the body, known as a peripheral vascular disease (PVD) (1). Costantino et al. (1) have discussed the attractive molecular targets in preventing PVD, an age-related pathology in the vasculature and the heart. They observed that environmental factors such as overnutrition, smoking, pollution, sedentary lifestyles, and genetic factors are involved in premature defects in mitochondrial functionality, insulin signaling, endothelial homeostasis, and redox balance. Several studies demonstrated the association of aging with the deterioration of cardiovascular homeostasis and metabolic disturbances, specifically insulin resistance (1). Furthermore, age plays an important role in the deterioration of cardiovascular functionality, resulting in an increased risk of CVD, such as hypertension, diabetes, and obesity (2–4). In addition, it has been reported that the onset and prevalence of CVD are dependent on sex differences, especially in older adults (3). Nonetheless, age is shown to be independently associated with inflammation and risk for CVD, which is reliant on health behaviors such as physical inactivity, poor nutrition, drinking alcohol, sugary beverages, and smoking (5). Given that CVD is continuously increasing in older patients, it is critically necessary to uncover the impact of nutrition on CVD in future clinical practice to improve outcomes in the older population.

## Why do cardiovascular diseases and SARS-CoV-2 have a significant impact?

Cardiovascular disease (CVD) is a significant problem in developed and developing countries (6). The increasing incidence of CVD in the past 25 years has become a significant concern for public health. The majority of underlying pathogenesis and progression associated with nearly all CVDs are caused by predominantly underlying atherosclerotic origin leading to conditions such as coronary artery disease, peripheral arterial disease, cerebrovascular disease, and venous thromboembolism (6). Many factors contribute to the development of cardiovascular diseases (CVD), such as unhealthy eating habits, lack of exercise, obesity, aging, gender, genetics, and smoking (6). A new viral infection called Coronavirus disease 2019 (COVID-19) is causing illness. It is caused by a severe acute respiratory syndrome coronavirus 2 (SARS-CoV-2; formerly known as 2019-nCoV). In clinical settings, several pathological evidence has been observed that strongly correlate with COVID-19 and peripheral vascular disease (PVD) (7). PVD causes the occlusion of the blood vessels outside the heart and brain. It is a well-established fact that nutrition and variations in food play an essential role in the development of CVD in COVID-19 patients (8, 9). Therefore, there is substantial evidence supporting the prevention of CVD progression through nutrition. Individuals who are at risk or have PVD with COVID-19 can develop an array of side effects, but nutrition can have positive impacts on their health, particularly from changes in their lifestyle (10). The present review article examined how nutrition status affects PVD progression and the severity of COVID-19- symptoms. Nutrition has been shown to affect the prevention of PVD and COVID-19 positively.

## Cardiovascular disease risk factors

Cardiovascular disease (CVD) is a major health concern worldwide, and the number of deaths caused by heart attacks and strokes is increasing every year (11). According to current research, over 70% of the population has multiple risk factors for developing CVD. Some of these risk factors can be controlled, such as high blood pressure, high blood cholesterol levels, smoking, diabetes, overweight or obesity, lack of physical activity, unhealthy diet, and stress. However, some risk factors cannot be controlled, such as age (risk increases with age), sex (men are at greater risk), family history, and race. Modifiable risk factors for CVD include preventing hypertension and abnormal lipid levels (11). It is important to note that a small percentage of the general population, ranging from two to 7 %, have no risk factors associated with CVD. Typically, these risk factors do not occur alone. Instead, having multiple risk factors increases the likelihood of developing the disease significantly. With just one risk factor, the probability of developing CVD is four-fold. However, if there are multiple risk factors present, the likelihood of developing the disease increases to 60-fold. Therefore, it is crucial to manage CVD risk factors to prevent heart disease and address the significant public health needs associated with this issue. Research indicates that there is a correlation between sleep and cardiovascular disease. Sleeping too little or too much can increase the risk of developing the disease. For most adults, sleeping less than 6 h or more than 9 h contributes to this risk. Modifiable risk factors are responsible for greater than 90 % of all cases of myocardial infarctions (11). Managing high-risk individuals to prevent CVD-related mortality, stroke, heart failure, and other adverse outcomes is crucial.

Studies suggest that treating multiple risk factors effectively is more beneficial in preventing CVD than solely focusing on modifying one risk factor (12, 13). This approach is particularly effective in treating individuals who are at high risk of developing CVD (11).

## The pathophysiological link between nutrition and peripheral vascular disease

Nutrition and food variations have been found to play a vital role in developing PVD and plaque build-up. There is a significant link between nutrition and PVD, which will be explored in this section. Consuming poor-quality diets, high in refined grains, added sugars, salt, unhealthy fats, and animal-source foods and low in whole grains, fruits, vegetables, legumes, fish, and nuts, can lead to PVD. These diets are often high in processed food products, which are typically packaged and ready to consume, and low in whole foods and freshly prepared dishes. Modern food environments contribute to unhealthy diets, which is a problem that is likely to become more widespread as food environments in low to middle-income countries shift to resemble those of high-income countries (14). To this end, unhealthy dietary patterns, such as the Western Diet, can lead to CVD by damaging the arterial/venous blood vessels, causing impediments to blood flow in the peripheral vascular system (15).

It is important to note that Western diets are usually high in saturated fats, added sugars, and sodium. Such diets can cause cholesterol and blood pressure levels to rise, leading to fatty acid build-up in arteries and veins. Conversely, Mediterranean diets can lower the risk of CVD by improving blood lipid profile (especially low-density lipoprotein cholesterol and triglycerides), reducing oxidation of lipids, and decreasing the risk of thrombosis (i.e., fibrinogen levels) (16). These diets contain plant-based foods, whole grains, nuts, seeds, essential fatty acids, fish oil, herbs, and spices (17). Additionally, they are rich in micronutrients like vitamins C, E, and D and significant minerals (18). Certain dietary patterns can either increase the risk of developing CVD or have protective effects in preventing and treating CVD and potentially PVD (Table 1) (6, 19, 20).

**Table 1:** Healthy eating plan (Myplate, 76).

Foods	Examples	Amount
Fruits and vegetables	Apple, orange, banana, mango	2 cups
Whole grains	Whole grain breads, pasta	6 oz
Low fat and non-fat proteins	Nuts, legumes, fish, seafood, lean poultry and meat	5 oz
Polyunsaturated fat	Avocado, coconut oil, olive oil, sunflower seed	11–22 gm
Low sodium, little to no added sugar, limit alcohol intake	Any processed/fried food	Not recommended

Although the link between CVD and nutritional factors has received a lot of attention in recent years, the relationship between nutrition and PVD has been overlooked. Recent studies have shed light on how food can improve the condition of PVD and have identified the significant nutrients that can promote good health in people with PVD. Results show that Omega-6, olive oil, and vitamin C interact at a biochemical level to lower risk factors (21), while fish oil, carnitine, and vitamin E are dietary therapies that have demonstrated clinical benefits for PVD patients (21).

In regard to PVD, the potential effect of fiber, folates, or vitamin B6 is only supported by epidemiological evidence (22). However, the available data is limited, and there is no convincing evidence to support the therapeutic efficacy of dietary therapies aimed at improving symptoms and reducing risk factors in PVD patients. Therefore, more research is undoubtedly necessary to improve the health conditions of individuals with PVD.

## Management of peripheral vascular disease via nutrition pathway

Peripheral vascular disease in those under 60 years old is considered premature. This condition is associated with increased cardiovascular morbidity, limb loss, and death (23). People with PVD require special attention to their diet and nutrition due to their advanced age, poor diet habits, and immobility that are associated with the disease process and co-morbid conditions. Because medical nutrition therapy is cost-effective, it is important to prioritize dietetics care in managing PVD. However, there is still much to be learned about how to optimize dietetic care for PVD. It is possible that the problem lies in the lack of nutritional education provided at medical schools (24). This is considered a failure of current medical education strategies. It is also concerning that fully trained dietitians display variations in practice and associated clinical impact due to lack of evidence base when treating patients with PVD. In conclusion, medical professionals should be aware of which high-risk factors lead to the development of premature PVD in patients less than 60 years. Improving disease detection rates and conducting routine screening of the Ankle-brachial Index should be practiced in high-risk patients (25).

## The interrelationship between nutrition, COVID-19, and peripheral vascular disease

The typical symptoms of COVID-19 commonly manifest with symptoms such as fever, dry cough, and fatigue, often concomitant with pulmonary involvement. In instances of severe manifestation, there is an observed elevation in levels of specific cytokines (e.g., IL-2, IL-7, IL-10), granulocyte colony-stimulating factor (G-CSF), monocyte chemotactic protein (MCP), and TNF-α. This inflammatory cascade and cytokine storm are pivotal in the development of severe acute respiratory syndrome and multi-organ failure, emphasizing the significance of the immune system in the disease progression (26, 27). Certain demographic groups with underlying conditions like obesity, hypertension, type 2 diabetes, ischemic heart disease, and chronic obstructive pulmonary disease are at greater risk of severe manifestations of COVID-19 (28–30). It is noteworthy that many of these chronic non-communicable diseases are intricately linked to dietary patterns and lifestyle characteristics. Moreover, high carbohydrate, saturated fat, and refined sugar diets can increase the risk of obesity. Abdominal obesity has been linked with the presence of oxidative stress and inflammation, which has been associated with decreased lung function (31). Therefore, a well-structured and nutritious diet is crucial for the efficient production of antibodies, as well as for the mitigation of oxidative stress and inflammatory conditions. This emphasizes the pivotal role of adequate protein, vitamins, minerals and omega-3 fatty acids intake in promoting a robust immune response (32).

The Mediterranean diet is known as one of the healthiest dietary patterns globally. It has been shown to have a preventive effect on cardiovascular diseases and type-2 diabetes in various trials (33). The diet primarily consists of plant-based foods such as fruits, vegetables, legumes, nuts, and olive oil, which are significant sources of bioactive polyphenols. Particularly, polyphenols help alleviate the immune response, increase antioxidant defenses, improve vascular reactivity, and decrease tissue inflammation and cell infiltration, thereby promoting metabolic and cardiovascular health (34). In summary, adhering to the Mediterranean diet has a positive impact on cardiovascular diseases and cardiometabolic disorders, such as diabetes, which can increase the risk of COVID-19 infection and related outcomes.

The U.S. Dietary Guidelines Advisory Committee has created three dietary patterns for better health and nutrition. These patterns are the Healthy-US type Pattern, the Healthy Mediterranean style Pattern, and the Healthy Vegetarian Pattern. All three components are associated with health benefits (35). Additionally, the Dietary Approaches to Stop Hypertension (DASH) diet has also been shown to improve cardiovascular risk factors. It emphasizes fruits, vegetables, whole grains, and low-fat dairy products while discouraging sugar-sweetened foods, beverages, red meat, and added sugars (36). The DASH diet has been linked to lower systolic blood pressure, diastolic blood pressure, and cholesterol (37). If individuals make healthier choices, the food systems will have a greater incentive to produce healthier options. On the other hand, a more nutritious food supply enables individuals to make healthier choices.

Developing food systems that promote healthier dietary patterns involves enhancing the food supply by producing more heart-healthy foods and fewer foods associated with CVD. This involves growing and raising these foods and ensuring that they are accessible and acceptable to everyone, including those with lower socioeconomic status. Furthermore, it is difficult to maintain a healthy diet in the modern global food system due to several reasons, such as the extensive distance between production and consumption, multiple and complex transformations in ingredients, and many other factors that discourage diversity and freshness. Additionally, there are political, economic, and environmental challenges to transforming the food system. Governments may not have the capacity to implement change (38, 39). However, it is possible to bring about change by educating people about how the modern food system shapes dietary patterns and eating behaviors. It is necessary to understand the relationship between food systems, food environments, diets, adverse public health, and environmental outcomes (40, 41). Better diets can lead to significant improvements in public health and environmental sustainability.

## Impact of dietary supplements and nutrition on risk of COVID-19

A recent study showed that those taking probiotics, omega-3 fatty acids, multivitamins, or vitamin D had a lower risk of SARS-CoV-2 infection (42). A randomized double-blinded control trial study on zinc supplementation showed that patients treated with zinc had a lower mortality rate than those not treated with zinc (43). Another randomized placebo-controlled study showed that taking vitamin C (500 mg twice daily) may reduce inflammation in hypertensive and/or diabetic obese patients by lowering IL-6 and C-reactive protein levels. The study suggests that vitamin C could be beneficial in treating severe cases of COVID-19 (44). Moreover, another systematic review has shown that vitamin D supplementation can be used as adjuvant therapy for COVID-19, as it effectively reduces its severity. However, further studies are needed to confirm these findings (45).

Most of the studies suggest that making simple changes to diet and lifestyle can significantly and rapidly reduce the risks associated with COVID-19 (46, 47). These changes include regular exercise, getting enough sleep, eating a plant-based diet with more fruits and vegetables, reducing the intake of sodium and red/processed meat, maintaining a healthy weight, taking dietary supplements, and spending time in nature (48–52). These interventions have been shown to benefit COVID-19 outcomes and overall health. It is important to communicate the power of these individual lifestyle changes to the public, especially when the benefits outweigh the risks. Embracing the lifestyle-centered approach could save many lives and prevent the need for vaccine mandates.

## During the COVID-19 lockdown, changes in dietary habits influenced the risk of CVD

The COVID-19 pandemic has had a varied impact on CVD risk and management. While some populations have improved their diet quality due to the shift toward home-cooked meals and reduced dining-out options, others have experienced poorer diet patterns. In general, government-imposed quarantines and the move toward a more private lifestyle have led to an increase in the consumption of home-cooked meals, which has moderately improved diet quality. However, this shift has also worsened food insecurity and stress-related unhealthy eating behavior, leading to higher intake of snacks and sweets (53, 54). A recent study conducted during the first wave of COVID-19 lockdown revealed that individuals in Canada, Mexico, Mediterranean countries, and Europe, with the exception of France, consumed more fruits and vegetables, and were more likely to adhere to the Mediterranean diet (53, 54). These findings suggest that regions with more diverse and healthy eating patterns may have lower COVID-19 and CVD risk. On the other hand, Latin and South American countries have shown an overall shift toward unhealthy eating (53, 54).

Furthermore, several worldwide studies have observed a universal increase in meal frequency, snacking, and overeating in addition to the improved adherence to Mediterranean-style diet principles (53, 54). During the lockdown, several studies were conducted to examine the impact of quarantine on people’s eating habits (53, 54). These studies suggest that although dietary choices may be healthier, there were more irregular eating patterns and a surplus of energy intake associated with quarantine. This may have been due to more sedentary behavior among both children and adults, which created an “obesogenic” environment that increases the risk for CVD and other metabolic disorders (55, 56).

It may also be possible that irregular eating patterns, such as increased snacking and alcohol consumption during the pandemic, stemmed from heightened stress, boredom, and loneliness caused by isolation. A study on obesity risk factors during the pandemic found a significant increase in depressive symptoms and mental illness globally, which can lead people to eat high sugar foods to improve and boost their mood and manage stress (57, 58). Particularly, people already living with mental illness, obesity, and experiencing loneliness during the pandemic are vulnerable to unhealthy eating habits that can lower diet quality and increase the risk of CVD (57, 58).

## During the pandemic, food insecurity is a social determinant for COVID-19 and CVD risk

The COVID-19 pandemic has exacerbated the pre-existing issue of food insecurity in communities across the USA, particularly affecting those with low socioeconomic status (59). Unemployment, panic buying, price inflation of staple foods, and interruptions in the food supply chain all contributed to a 32% increase in food insecurity during the initial months of the pandemic (60). Those who faced food insecurity were more likely to consume fewer fresh fruits and vegetables and more fast food, which is typically ultra-processed and of poor dietary quality. This can have a detrimental effect on the immune system and increase the risk of CVD (58). Fluctuating food availability can lead to irregular eating patterns and periods of deprivation, leading to insulin resistance, elevated blood glucose levels, increased blood pressure, and fat accumulation, all of which are known risk factors for CVD (59).

## Nutritional therapy and COVID-19

According to World Health Organization (WHO), it is estimated that more than 700 million people worldwide have contracted the SARS-CoV-2 virus (61). Although only a small fraction of those infected required admission to the intensive care unit, many needed nutritional therapy to support their recovery (61). Since the virus is highly contagious, it is crucial to provide excellent nutrition care to healthcare providers while minimizing exposure.

The American Society for Parenteral and Enteral Nutrition (ASPEN) has issued recommendations for optimizing nutritional therapy in critically ill COVID-19 patients. COVID-19 can trigger an extreme hyperinflammatory response known as cytokine storm syndrome (62). To manage inflammation, there are specific recommendations for nutritional therapy based on critical care nutrition intervention principles, adjusted for the limitations of the contagious SARS-CoV-2 disease. It is important to note that a registered dietitian should be responsible for ensuring nutrition safety while administering supplemental care to improve the patient’s health.

According to a study conducted by Patel et al. (61), positive health outcomes have been observed in patients who switch from enteral nutrition (EN) to parenteral nutrition (PN) at a lower threshold (61). This recommendation helps the gut function as a promoter in counteracting dysbiosis, regulating immune responses, and restoring anti-inflammatory processes (63–67). Furthermore, the study suggests that critically ill COVID-19 patients benefit from EN as the preferred method of nutrition delivery, as it improves the health of their gut.

In summary, EN is preferred over PN. Additionally, it is also recommended to supplement with protein, prebiotic fiber, and probiotics daily, while following the Mediterranean diet principles. This approach may be a strategic way to address both short- and long-term conditions associated with COVID-19 infection and to improve mortality rates and the overall well-being of affected populations (61).

## Impact of COVID-19 and PVD

It has been observed in clinical settings that COVID-19 can trigger a coagulation response which leads to deep vein thrombosis (DVT) and pulmonary embolism (PE) as well as thrombosis of microcirculation and disseminated intravascular coagulation (DIC). A study has reported an increase in pro-coagulant profile, such as IL-6, which induces tissue factor expression, high levels of fibrinogen, and d-dimer, which are markers of the severity of COVID-19, as well as prolongation of activated partial thromboplastin time (aPTT) (68). The mechanisms by which the COVID-19 virus induces/complicates thromboembolic events are still under investigation.

Older adults, minorities (Hispanic and African Americans), those over the age of 50, overweight, with abnormal cholesterol, history of cerebrovascular disease and stroke, diabetes, family history of high blood pressure, and kidney disease are at risk for PVD (69, 70). Diet can play a role in why minorities over 50 fall into the at-risk category. For instance, the traditional Hispanic diet is heavily influenced by the community and traditional dietary patterns of their countries and locations. Despite their diverse ancestral histories, traditional Hispanic cuisines depend on grains, carbohydrates, and beans with the incorporation of fresh fruits and vegetables in their diet. However, according to the U.S. Department of Agriculture (USDA), acculturation integration and the changing nature of Hispanic cuisine have significant consequences on Hispanic health (71). Hispanic Americans consume more saturated fats, refined sugars, and grains, and meals away from home than traditional family-cooked meals. Nutrition education programs designed to improve the quality of the Hispanic diet are presently driven by a combination of preserving healthy traditional Hispanic diet elements, such as reliance on beans, rice, and tortillas, fresh fruits and vegetables, and changing other eating habits, such as lessening fast food consumption, reduced consumption of high-fat dairy products and less use of fat in cooking (72). All older adults should use the MyPlate plan, a government-funded customizable food plan based on age, sex, height, weight, and physical activity level. According to MyPlate, older adults should consume an individualized caloric plan to maintain a healthy lifestyle and follow a heart-healthy diet, refer to (Table 2). For a 2000 calorie diet, individuals should consume 2 cups of fruit, 2 ½ cups of vegetables, 6 ounces of whole grains, 5 ounces of protein foods, and 3 cups of low-fat dairy while incorporating healthy fats. Polyunsaturated fats like omega-3 have anti-inflammatory properties, and omega-6, which can regulate metabolism, are fats healthy for the heart (Tables 2–4) (73). It has been found that lifestyle choices such as physical inactivity, smoking, and drug use (7), when combined with poor diet, can increase the risk of PVD. According to a study, *“The Role of Physical Activity and Sedentary Behaviors in Explaining the Association between Acculturation and Obesity among Mexican American Adults,”* physical activity related to work and transportation decreases as foreign-born Hispanics become more acculturated to U.S. culture, leading to a more sedentary lifestyle. This may explain why obesity outcomes are worse among more acculturated people. However, it is not yet clear how different forms of physical activity and sedentary habits affect the relationship between acculturation and obesity.

**Table 2:** Nutrition examples of heart healthy eating plan (Myplate 76).

Fruits	Vegetables	Whole grains	Protein	Low-fat dairy	Healthy fat
2 cups	2 ½ cups	6 oz	5 oz	3 cups	Omega-3 Omega-6

**Table 3:** Physical activity essential to healthy aging (CDC 75).

Duration	Type	Activity
150 min/week	Aerobics	Moderate intensity activity (Brisk walking: 30 min/day; 5X week)
2 days/week	Strengthen muscles	Weightlifting
3 days/week	Improve balance	Standing on one foot

**Table 4:** Specific heart healthy micronutrients (per day).

Micronutrients	Animal food sources	Plant food sources	RDA for women	RDA for MeN
Vitamin C (Ascorbic acid)	Eggs, raw liver, fish, fish roe	Lime, mandarin, orange, kiwi, kale, blackcurrants	75 mg	90 mg
Vitamin D (1, 25 Dihydroxy cholecalciferol)	Dairy, meat, eggs	Mushrooms	15 μg	15 μg
Vitamin E (alpha tocopherol)	NA	Almonds, sunflower, peanuts, hazelnut, soybeans	15 mg	15 mg
Zinc	Oyster, red meat, poultry	Lentils, beans, chickpeas, whole grains	10 mg	15 mg
Flavonoids	NA	Citrus fruits, berries, apples, onion, leafy vegetables, green tea, wines	250–400 mg	250–400 mg

The risk of severe illness from COVID-19 is higher among minorities, especially Hispanics and African Americans; older individuals come under a more significant risk of infection (CDC, 2020). It has been reported that more clinical studies are being conducted on the development of arterial complications in severely ill COVID-19 patients. There is increasing anecdotal evidence that COVID-19 patients are experiencing a more significant burden of coronary thrombosis. Additionally, case reports have shown occurrences of acute aortic and limb arterial thrombosis in COVID-19 patients, with varying outcomes (74). This provides further support for the increased thrombotic risk of SARS-CoV-2 infection. Patients who are severely ill with COVID-19 may require intensive care or a ventilator to assist them with breathing (CDC, 2020) (75). Additionally, individuals with certain underlying medical conditions, regardless of risk, are at a greater risk of developing severe disease from SARS-CoV-2 infection (CDC, 2020) (75). Studies have shown that those who are elderly, critically ill, underrepresented minorities, or have underlying medical conditions are at the highest risk of contracting the virus and experiencing worsened symptoms.

The impacts of nutrition on susceptibility to COVID-19 and PVD have long-term consequences. Recent studies have shown that the Western Diet (rich in saturated fats, sweets, and refined carbohydrates) contributes to the global prevalence of obesity, cardiovascular disease, type 2 diabetes, and the risk of COVID-19 infection (10). Additionally, consumption of the Western diet weakens the adaptive immune response while strengthening the innate immune system, resulting in endothelial dysfunction and an uncontrolled inflammatory cascade (10). Furthermore, COVID-19 triggers peripheral inflammation, leading to long-term consequences in individuals who have already been exposed to the virus and cannot return to a healthy baseline. Poor management of nutrition exacerbates the condition, making it crucial to prioritize the availability of nutritious foods for individuals at risk of PVD and COVID-19 (10, 77–80).

## COVID-19 association with underlying conditions of PVD

A recent study has shown that COVID-19 can severely impact individuals with underlying health conditions such as diabetes and hypertension, especially in relation to peripheral arterial complications (81). Previous studies have identified several risk factors for COVID-19, such as age, diabetes, smoking, obesity, and hypertension, which are also associated with atherosclerosis. However, a note of the studies have implicated pre-existing PVD in the severity of the COVID-19 infection or outcome, except for cerebrovascular disease (7) This leads us to hypothesize that COVID-19 may worsen pre-existing PVD and limb ischemia by directly targeting the endothelium or increasing blood viscosity and expression of coagulation factors such as von Willebrand Factor (vWF), fibrinogen, and d-dimers. Additionally, the cytokine storms evoked by the viral infection may lead to the rupture of atherosclerotic plaque, which can attract circulating platelets and form a thrombus, further worsening PVD (82). This leads to the rupture of plaque and the ability to attract circulating platelets at the site of the injury and form the thrombus (83). Regarding chronic venous diseases, particularly pre-existing PVD, COVID-19 infection may worsen clinical symptoms or even create new thrombosis. Therefore, finding optimal therapeutic agents to manage COVID-19 disease is urgently needed.

## Conclusion and future directions

The ongoing research on the SARS-CoV-2 and subsequent COVID-19 pandemic continues to yield new findings regarding their impact on human well-being and their implications for the forthcoming years. The global focus efforts have been directed not only at improving and enhancing vaccines against the virus but also at crafting increasingly targeted and specific medical therapies. It is evident that comprehending the mechanisms of the virus’s action, particularly the excipients or supplements that can modulate the viral inflammatory process, constitutes a fundamental cornerstone in the fight against COVID-19. In this context, dietary interventions, achieved through the consumption of essential macronutrients and micronutrients, serve as the primary fortification in both preventing and combating the virus and its correlated symptoms, irrespective of hospitalization. Furthermore, nutrition assumes the role of personalized medicine contingent upon the patient’s specific condition. Based on these assessments, the attributes and requisites of the immune system undergo modification, commensurate with the capacity to respond to the infection and modulate its propagation at a multi-organ level. This underscores the adaptability and reshaping of dietary regimens as necessitated by personalized assessments and directives predicated on the state of health of COVID-19-positive patients.
